# Impact of PET Reconstruction on Amyloid-β Quantitation in Cross-Sectional and Longitudinal Analyses

**DOI:** 10.2967/jnumed.123.266188

**Published:** 2024-05

**Authors:** Gihan P. Ruwanpathirana, Robert C. Williams, Colin L. Masters, Christopher C. Rowe, Leigh A. Johnston, Catherine E. Davey

**Affiliations:** 1 Department of Biomedical Engineering, University of Melbourne, Melbourne, Victoria, Australia;; 2 Melbourne Brain Centre Imaging Unit, University of Melbourne, Melbourne, Victoria, Australia;; 3 Florey Institute of Neurosciences and Mental Health, University of Melbourne, Melbourne, Victoria, Australia;; 4 Australian Dementia Network, Melbourne, Victoria, Australia; and; 5 Department of Molecular Imaging and Therapy, Austin Health, Melbourne, Victoria, Australia

**Keywords:** amyloid-β, reconstruction parameters, [^18^F]florbetapir, [^18^F]flutemetamol, PSF, TOF

## Abstract

Amyloid-β (Aβ) accumulation in Alzheimer disease (AD) is typically measured using SUV ratio and the centiloid (CL) scale. The low spatial resolution of PET images is known to degrade quantitative metrics because of the partial-volume effect. This article examines the impact of spatial resolution, as determined by the reconstruction configuration, on the Aβ PET quantitation in both cross-sectional and longitudinal data. **Methods:** The cross-sectional study involved 89 subjects with 20-min [^18^F]florbetapir scans generated on an mCT (44 Aβ-negative [Aβ−], 45 Aβ-positive [Aβ+]) using 69 reconstruction configurations, which varied in number of iteration updates, point-spread function, time-of-flight, and postreconstruction smoothing. The subjects were classified as Aβ− or Aβ+ visually. For each reconstruction, Aβ CL was calculated using CapAIBL, and the spatial resolution was calculated as full width at half maximum (FWHM) using the barrel phantom method. The change in CLs and the effect size of the difference in CLs between Aβ− and Aβ+ groups with FWHM were examined. The longitudinal study involved 79 subjects (46 Aβ−, 33 Aβ+) with three 20-min [^18^F]flutemetamol scans generated on an mCT. The subjects were classified as Aβ− or Aβ+ using a cutoff CL of 20. All scans were reconstructed using low-, medium-, and high-resolution configurations, and Aβ CLs were calculated using CapAIBL. Since linear Aβ accumulation was assumed over a 10-y interval, for each reconstruction configuration, Aβ accumulation rate differences (ARDs) between the second and first periods were calculated for all subjects. Zero ARD was used as a consistency metric. The number of Aβ accumulators was also used to compare the sensitivity of CL across reconstruction configurations. **Results:** In the cross-sectional study, CLs in both the Aβ− and the Aβ+ groups were impacted by the FWHM of the reconstruction method. Without postreconstruction smoothing, Aβ− CLs increased for a FWHM of 4.5 mm or more, whereas Aβ+ CLs decreased across the FWHM range. High-resolution reconstructions provided the best statistical separation between groups. In the longitudinal study, the median ARD of low-resolution reconstructed data for the Aβ− group was greater than zero whereas the ARDs of higher-resolution reconstructions were not significantly different from zero, indicating more consistent rate estimates in the higher-resolution reconstructions. Higher-resolution reconstructions identified 10 additional Aβ accumulators in the Aβ− group, resulting in a 22% increased group size compared with the low-resolution reconstructions. Higher-resolution reconstructions reduced the average CLs of the negative group by 12 points. **Conclusion:** High-resolution PET reconstructions, inherently less impacted by partial-volume effect, may improve Aβ PET quantitation in both cross-sectional and longitudinal data. In the cross-sectional analysis, separation of CLs between Aβ− and Aβ+ cohorts increased with spatial resolution. Higher-resolution reconstructions also exhibited both improved consistency and improved sensitivity in measures of Aβ accumulation. These features suggest that higher-resolution reconstructions may be advantageous in early-stage AD therapies.

## Visual Abstract

**Figure:**
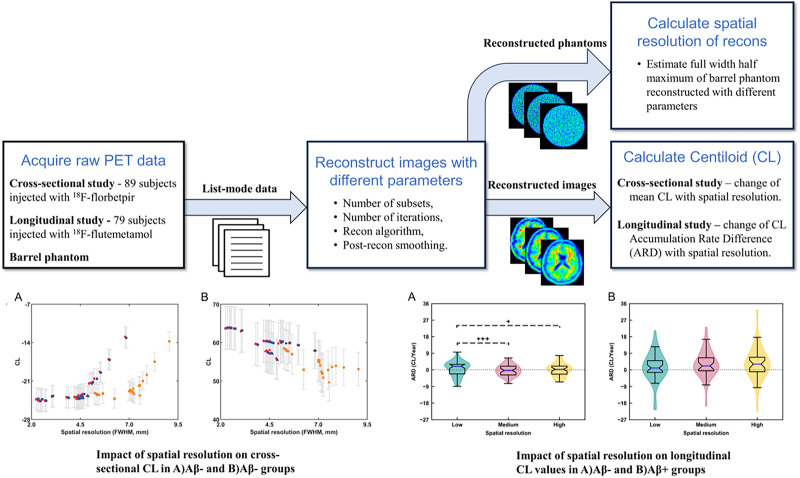

Amyloid-β (Aβ) accumulation in the brain is a pathologic indicator of Alzheimer disease (AD) that can be imaged using PET. Second-generation PET radiotracers, such as [^18^F]florbetaben, [^18^F]florbetapir, and [^18^F]flutemetamol, have been designed for on-target binding of Aβ plaques, enabling better diagnosis, management, and treatment of AD patients (1*,*^2^). The extent of Aβ PET deposits is most commonly measured using scaled variants of the SUV, such as the SUV ratio (SUVR) and the centiloid (CL) scale (2*,*^3^).

PET imaging has a low spatial resolution relative to other imaging modalities because of factors including the positron range of the radioisotope, photon scattering, and hardware-specific limitations (4). The low resolution renders PET imaging particularly susceptible to the partial-volume effect (PVE), by which quantitative PET metrics are degraded by the presence of multiple tissue types within a single voxel (5). Although the highest achievable spatial resolution of the PET image is determined by the scanner hardware, reconstruction algorithms and associated parameters are often chosen to provide lower resolution to maintain spatial noise variance at clinically accepted values (6). The ordered-subset expectation maximization (OSEM) algorithm, characterized by the numbers of subsets and iterations, produces reconstructed images that are prone to increased spatial noise variance at high iteration numbers (7); this can be reduced by early termination of the iterative loop and postreconstruction smoothing, at the cost of reduced spatial resolution and increased PVE (6–8).

The noise and spatial resolution of PET images have been improved by the use of time-of-flight (TOF) and point-spread function (PSF) information in the reconstruction process (9). TOF incorporates the difference in detector arrival time between 2 photons into the reconstruction process, enabling both faster reconstruction convergence and reduced image noise (10). The PSF was introduced to improve the spatial resolution of PET images by incorporating the scanner response resolution into the reconstruction algorithm (4*,*^11^*,*^12^). In altering the quality of PET images, these reconstruction improvements impact the quantitative metrics derived from the resultant images (11–13).

Studies have examined the impact of the reconstruction algorithm and associated parameter configurations on tumor PET quantitation (11–13). However, few studies have considered the effect of reconstruction on neuroimaging PET quantitation. These studies were based primarily on phantom scans, including only a small patient cohort (14–16). A notable exception is a study examining the impact of reconstruction parameters on [^18^F]FDG and [^18^F]flutemetamol scans of AD subjects (17). This cross-sectional study showed a significantly lower Aβ SUVR in the control group with PSF-enabled reconstruction than with OSEM reconstruction. Conversely, the AD group showed no significant differences in Aβ SUVR with PSF-enabled reconstruction. The study used a limited variety of reconstructions, modifying the algorithm but not the associated parameter settings. Studies examining the impact of reconstruction on longitudinal Aβ metrics are notably absent from the literature.

To explore the effect of PET reconstruction on longitudinal Aβ PET measures in AD, it is necessary to establish an expectation of how measures will change over time. Aβ accumulation is known to follow a sigmoidal pattern over decades of Aβ deposition (18). However, for studies of relatively short duration, a linear Aβ accumulation rate can be assumed (18*,*^19^). This assumption facilitates an examination of the effect of reconstruction protocols on Aβ quantitation over short periods.

We examined the impact of PET image spatial resolution, as determined by the choice of reconstruction algorithm and associated parameters, on Aβ CL measures of AD progression. Both cross-sectional and longitudinal Aβ PET datasets were reconstructed using a range of configurations and were evaluated according to their impact on cross-sectional Aβ CL and separation of CL between Aβ-negative (Aβ−) and Aβ-positive (Aβ+) groups, as well as according to their impact on the estimated rate of Aβ accumulation over time.

## MATERIALS AND METHODS

The PET/CT specifications, subject demographics, and scanning information are detailed in the methods sections of the supplemental materials (available at http://jnm.snmjournals.org).

### Image Reconstruction

#### Spatial Resolution Calculation

A cylindric phantom was used to measure the axial and radial spatial resolution achieved by a given reconstruction protocol by calculating the full width at half maximum (FWHM) in both directions, as proposed by Lodge et al. (8). More details are provided in the supplemental materials.

#### Cross-Sectional Study

Each subject’s data were reconstructed using a set of protocols, each defined by the algorithm and parameter configuration, and chosen to cover a range of spatial resolutions. The FWHM associated with each protocol was calculated using the cylindric phantom. Three reconstruction algorithms were used: ordinary Poisson OSEM (OP), OSEM + TOF (OPTOF), and OSEM + TOF + PSF (PSFTOF), each with 2 parameters: number of OSEM iterations and number of subsets. Possible values for subsets and iterations differed between algorithms because of limitations of the scanner software. OP, OPTOF, and PSFTOF were run with 1 of 6 possible iterations, iterations∈{2,4,6,8,10,12}, with 21 subsets for OPTOF and PSFTOF and 24 subsets for OP. Additional OP reconstructions were performed using iterations∈{4,6,8,10,12} with 4 subsets. Postreconstruction gaussian filters of 0, 1, and 5 mm were applied. A given reconstruction protocol was defined by the algorithm, number of subsets, number of iterations, and postreconstruction smoothing size. In total, 69 reconstruction configurations were applied per dataset.

#### Longitudinal Study

To evaluate the effect of spatial resolution on longitudinal Aβ PET quantitation, each subject’s scan was reconstructed with 3 distinct resolution categories: low (FWHM, 7.05 mm), medium (FWHM, 4.55 mm), and high (FWHM, 3.05 mm). The 7.05 mm FWHM low-resolution reconstruction was implemented using OP (4 iterations, 4 subsets, 2 mm postreconstruction gaussian smoothing); the 4.55 mm FWHM medium resolution, using OPTOF (4 iterations, 21 subsets, 0 mm postreconstruction gaussian smoothing); and the 3.05 mm FWHM high-resolution configuration, using PSFTOF (4 iterations, 21 subsets, 0 mm postreconstruction gaussian smoothing).

### Data Analysis

#### FWHM Variation

Each reconstruction protocol is associated with a specific spatial resolution, determined by estimating FWHM from the reconstructed barrel phantom. Supplemental Figure 1 shows the FWHM of each reconstruction protocol. Spatial resolution was calculated as a function of algorithm convergence, indicated by the product of subsets and iterations.

#### Cross-Sectional Study

Each image reconstruction was uploaded to CapAIBL (http://milxcloud.csiro.au) to generate Aβ SUVRs, estimated by calculating tracer retention inside a neocortical mask of brain regions with a known Aβ accumulation, such as frontal cortex, parietal cortex, and temporal cortex (20). The whole cerebellum was used as the reference region to generate the SUVRs. The Aβ positivity of the subjects was determined visually by an expert, separating subjects into Aβ− and Aβ+ groups. SUVRs were used in the cross-sectional study, converted to CLs using the CapAIBL conversion formula (20). In this article, the term *CL* denotes the Aβ CL generated by the CapAIBL software.

The effect of reconstruction on CL was examined by observing changes in mean CL as a function of both spatial resolution, quantified by FWHM, and convergence of the reconstruction, indicated by subsets × iterations, in both the Aβ− and the Aβ+ groups.

The impact of postreconstruction smoothing on CL was analyzed by calculating the mean pairwise CL difference between reconstructions with the same iteration and subset parameters but with differing postreconstruction smoothing, of either 5 or 0 mm. This was evaluated separately across Aβ− and Aβ+ cohorts. The effect size of mean CL separation between the Aβ− and Aβ+ groups was calculated using Cohen *d* as a function of the FWHM associated with reconstruction configurations.

To visualize the impact of spatial resolution on the separation between Aβ− and Aβ+ groups, the reconstruction protocol with the largest effect size (group separation) was compared with a typical clinical reconstruction protocol using OP with 4 iterations, 4 subsets, and 5 mm postreconstruction gaussian smoothing. For each reconstruction protocol, The CLs of both the Aβ− and the Aβ+ groups were tested for gaussianity and subsequently fitted with gaussian distributions, enabling a comparison of CL group separation between the 2 reconstruction protocols, via a *t* test. The mean CLs of high-resolution reconstruction in the Aβ− and Aβ+ groups were compared with the relevant mean CLs of clinical reconstructions using right-tailed and left-tailed *t* tests, respectively. One-tailed *t* tests were used, as it was hypothesized that the mean CL of the high-resolution reconstruction would be smaller than that of the clinical reconstruction in the Aβ− group and that the mean CL of the high-resolution reconstruction would be greater than that of the clinical reconstruction in Aβ+ group. A left-tailed F test was used to compare the CL variances of high-resolution reconstructions in both the Aβ− and the Aβ+ groups with the respective CL variance of the clinical reconstruction configuration under the hypothesis of greater CL variance in high-resolution reconstructions than in clinical reconstructions.

The performance of CL as a classifier between the Aβ− and Aβ+ cohorts was compared for the reconstruction configuration with the largest effect size and the clinical reconstruction. Receiver-operating-characteristic curves were made for both of the classifiers, and performance was summarized by calculating the area under the curve.

#### Longitudinal Study

To ascertain the effect of PET reconstruction on longitudinal Aβ quantitation, CLs were used to measure global Aβ PET uptake, estimated by uploading each reconstructed image volume to CapAIBL. Subjects were allocated into Aβ− and Aβ+ groups, with the Aβ positivity of a subject being determined using the CL estimate of the low-resolution OP reconstructed baseline scan, with a threshold CL of at least 20 (Supplemental Table 1).

On the basis of the known properties of the Aβ accumulation process, over a 10-y interval a linear Aβ accumulation rate can be assumed, resulting in a consistent accumulation across the 10-y period (19). Since there were 3 scans per subject within 5 y, well within the 10-y duration of linear Aβ accumulation, Aβ accumulation rates in both the first interval and the second interscan interval were computed and tested for conformity with the linearity assumption. Indeed, the linearity of the Aβ accumulation process was used to derive 2 surrogate metrics to analyze the impact of reconstruction configurations on longitudinal Aβ CL quantitation.

The first surrogate metric was the Aβ rate difference (ARD) between the second and first intervals for each subject and each reconstruction configuration. Given a linear Aβ accumulation assumption over the longitudinal study period, an ARD of 0 indicated a consistent accumulation rate over the 3 time points. The Wilcoxon signed-rank test was used to test whether the median ARD of each reconstruction was statistically significantly different from zero in both the Aβ− and the Aβ+ groups; for low- and high-resolution reconstruction configurations, right-tailed Wilcoxon signed-rank tests were used in both Aβ groups, and for the medium resolution configuration, left- and right-tailed Wilcoxon signed-rank tests were used in the Aβ− and Aβ+ groups, respectively. We decided on the use of right- or left-tailed Wilcoxon signed-rank tests depending on the sign of the median ARD. A right-tailed Mann–Whitney *U* test was also performed on both the Aβ− and the Aβ+ groups to test whether the higher-resolution reconstructions’ median ARDs were less than those of lower-resolution reconstructions.

For each subject and for each of the 3 reconstruction choices, a linear model was fitted across the 3 time points. Wilcoxon signed-rank tests were performed to test whether the median accumulation slopes of the fitted linear models were statistically significantly different from zero, as it was an indication of the number of generated positive slopes; right-tailed Wilcoxon signed-rank tests were used across all the reconstructions in both Aβ groups, except for the low-resolution reconstruction configuration in the Aβ− group, which used a left-tailed Wilcoxon signed-rank test. The choice of test was based on the sign of the median accumulation slope. The median accumulation slopes of the 3 reconstruction configurations were also compared using the left-tailed Mann–Whitney *U* test under the hypothesis that the median accumulation slopes of the higher-resolution reconstructions were greater than those of lower-resolution reconstruction. All tests were performed on the Aβ− and Aβ+ groups separately.

In addition to these statistical tests, the number of Aβ accumulators was calculated as a third comparison measure of reconstruction configurations. The study used 2 slope thresholds, 0 and 2 CL/y, to identify Aβ accumulators in both the Aβ− and the Aβ+ groups.

## RESULTS

### Cross-Sectional Study

**FIGURE 1.**
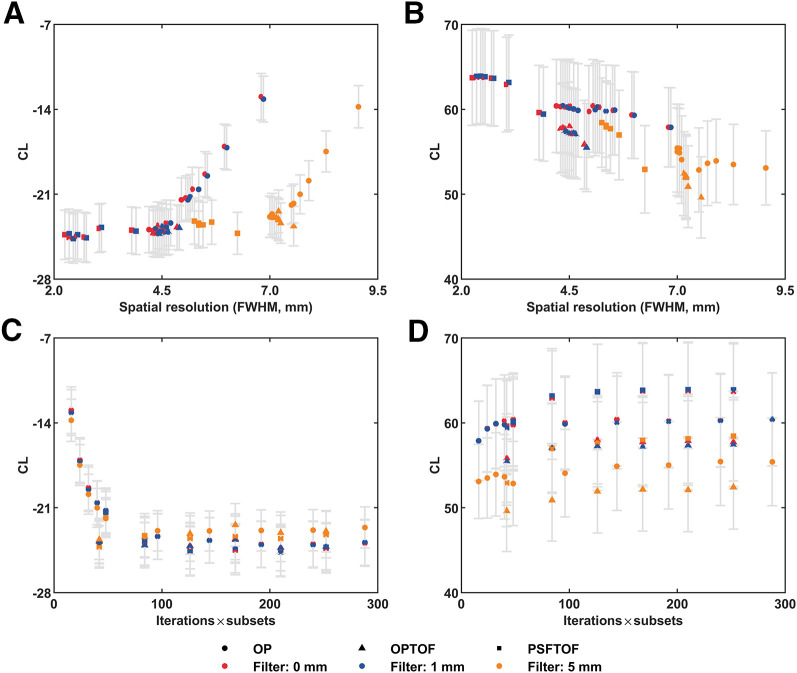
Impact of reconstruction, including spatial resolution and reconstruction convergence, on Aβ CL. (A and B) Impact of spatial resolution (FWHM), determined by reconstruction parameters, on mean Aβ CL in Aβ− (A) and Aβ+ (B) groups. Noticeable shift between red/blue and orange markers in B indicates that postreconstruction smoothing may impact mean CL for Aβ+ cohort. (C and D) Impact of convergence, determined by iterations × subsets, on mean Aβ CL in Aβ− (C) and Aβ+ (D) groups. Each data point represents mean Aβ CL across all subjects in group reconstructed with same protocol, defined by reconstruction algorithm, number of iterations, subsets, and postreconstruction gaussian smoothing. Spatial resolution of group was calculated as FWHM of cylindric phantom reconstructed with same protocol (8). Filter value in legend denotes FWHM of postreconstruction gaussian smoothing. Whiskers indicate SE.

CLs in both the Aβ− and the Aβ+ groups were impacted by the spatial resolution of the reconstruction configuration (Fig. 1). The standard errors of CLs in the Aβ− group were small compared with the Aβ+ group for all reconstructions (Figs. 1A and 1B). In the Aβ− group, no notable CL differences were seen across low FWHM reconstructions with 0 and 1 mm smoothing; CLs started to increase at an FWHM of approximately 4.5 mm (Fig. 1A). Reconstructions with 5 mm smoothing showed the same trend but with a plateaued region at an FWHM of 5.2–7 mm, followed by an increase in mean CLs. Application of postreconstruction smoothing shifted the no-smoothing reconstruction data to the right without a notable CL difference (Fig. 1A). In the Aβ+ group, there was no plateaued CL region as in the Aβ− group, with CLs decreasing across the full range of FWHMs (Fig. 1B).

Figure 1 shows mean CLs with varying postreconstruction smoothing filters applied to each reconstruction configuration. For the Aβ− cohort, mean CL appears to remain constant, despite the consequent shift in FWHM due to postreconstruction smoothing, with each dataset showing the same range and trend (Fig. 1A). However, Figure 1B indicates that postreconstruction smoothing may impact mean CL for the Aβ+ cohort. This issue is examined in more detail in the section on the smoothing effect on Aβ quantitation in the supplemental materials.

CLs in both the Aβ− and the Aβ+ groups were impacted by reconstruction convergence, depicted by iteration × subsets, and determined by parameter settings (Figs. 1C and 1D). In the Aβ− group, CLs decreased as convergence increased, plateauing at a CL of approximately 42. This trend was consistent irrespective of the extent of postreconstruction smoothing (Fig. 1C). There were no notable differences in CLs between the PSFTOF and OPTOF algorithms in the Aβ− group. However, only the OP reconstruction method allowed for iteration updates below 42, which led to an increase in mean CL.

In the Aβ+ group, the CLs decreased with the application of postreconstruction smoothing for all 3 reconstruction algorithms (OP, OPTOF, and PSFTOF). At each level of smoothing, CLs exhibited a slight increase with convergence for all 3 algorithms (Fig. 1D). Moreover, across all iteration × subsets, PSF reconstructions yielded the highest mean CL, followed by OP, and then TOF (Fig. 1D).

**FIGURE 2.**
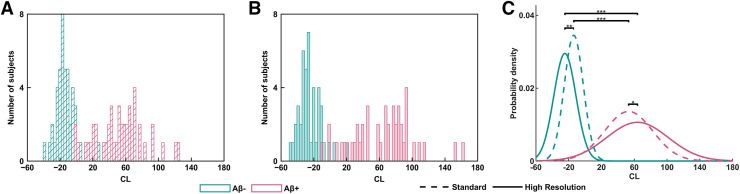
(A and B) Separation between Aβ− and Aβ+ groups using clinical reconstruction configuration OP (4 iterations, 4 subsets) with 5 mm smoothing (A) and high-resolution reconstruction configuration PSFTOF OP (10 iterations, 21 subsets) with 1 mm smoothing (B) resulted in largest separation between Aβ− and Aβ+ cohorts. (C) Fitted gaussian distributions on CLs of both reconstruction configurations are overlaid for ease of visualization. *Trend toward significant difference in mean (*P* = 0.07) and variance (*P* = 0.05). **Significant difference in mean (*P* = 0.00006) and trend toward significant difference in variance (*P* = 0.15). ***Significant difference between group means (*P* < 0.00005).

The separation between Aβ− and Aβ+ groups was dependent on the resolution of the reconstruction configuration. The difference in separation between Aβ+ and Aβ− groups with reconstruction configuration is shown in Figure 2 using a standard clinical reconstruction and a high-resolution reconstruction, resulting in the largest separation between the cohorts (described in the section on Cohen analysis in the supplemental materials). Although both the standard clinical reconstruction and the high-resolution reconstruction separated the Aβ+ and Aβ− groups significantly (*P* < 0.00005), the latter increased the dynamic range of CLs in both groups (Fig. 2C); the mean CL of the high-resolution reconstruction was significantly smaller (*P* = 0.00006) than the standard reconstruction in Aβ− group, and the mean CL of the Aβ+ group trended toward a significantly larger value (*P* = 0.07) in the high-resolution reconstruction than in the standard reconstruction. Moreover, the CL variances trended toward being significantly larger for the high-resolution reconstruction than for the standard reconstruction in both the Aβ− (*P* = 0.15) and the Aβ+ (*P* = 0.05) groups.

To compare the classification performance of the reconstruction configurations depicted in Figure 2, areas under the curve were calculated for the receiver-operating-characteristic curves of each. Areas under the curve did not notably differ between the reconstructions, with values of 0.989 and 0.992 for the clinical reconstruction and high-resolution reconstruction, respectively.

### Longitudinal Study

**FIGURE 3.**
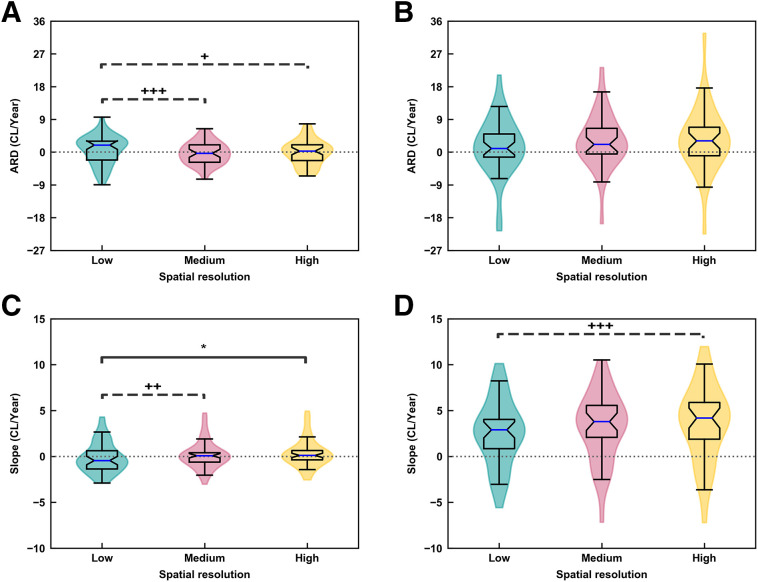
(A and B) ARDs between second and first time intervals for 3 reconstructions: low-resolution OP (4 iterations, 4 subsets, 2 mm smoothing, FWHM of 7.05 mm), medium-resolution OPTOF (4 iterations, 21 subsets, 0 mm smoothing, FWHM of 4.55 mm), and high-resolution PSFTOF (4 iterations, 21 subsets, 0 mm smoothing, FWHM of 3.05 mm) in Aβ− (A) and Aβ+ (B) groups. (C and D) Aβ accumulation slopes of longitudinal linear models for Aβ− (C) and Aβ+ (D) groups. Solid lines indicate significant median difference between groups, and dashed lines indicate trend toward significant median difference. *P = 0.03. ^+^*P* = 0.08. ^++^*P* = 0.07. ^+++^*P* = 0.06. High- and medium-resolution reconstructions of Aβ+ group show average increase in CL/y of 1.05 and 0.70, respectively, over low-resolution reconstruction group.

The consistency of longitudinal measurements of Aβ accumulation was analyzed across reconstruction configurations by analyzing the Aβ ARDs between the 2 periods, with an ARD of 0 denoting the best level of consistency (Figs. 3A and 3B). The data underlying these results are summarized in Supplemental Figures 4 and 5. In the Aβ− group, the median ARD of low-resolution reconstructed data trended toward a statistically significant value greater than zero (*P* = 0.1), and the median ARDs of both the medium- and the high-resolution reconstructions were not significantly different from zero. Higher-resolution reconstructions reduced the average CLs of the Aβ− group by 12 points. In contrast, in the Aβ+ group, the median ARDs of medium-resolution (*P* = 0.006) and high-resolution (*P* = 0.004) reconstructions were significantly greater than zero, and the median ARD of low-resolution reconstructions trended toward a value significantly greater than zero (*P* = 0.08). Consequently, medium- and high-resolution reconstructions resulted in more consistent Aβ PET longitudinal data than did low-resolution reconstructions, especially in the Aβ− group.

The median ARDs were dependent on the resolution of the reconstruction configuration. In the Aβ− group, the median ARD trended toward a significantly larger value for low-resolution data than for medium- (*P* = 0.06) and high-resolution (*P* = 0.08) data (Fig. 3A). However, significant changes in ARDs were not observed across the 3 reconstructions in the Aβ+ group (Fig. 3B).

Slopes of linear models fitted to the longitudinal data were impacted by the spatial resolution of the reconstruction (Figs. 3C and 3D). In the Aβ− group, the median slope of the low-resolution reconstructions was negative and trended toward a significantly nonzero value (*P* = 0.1), whereas the median slope of the high-resolution reconstructions was positive and trended toward a significantly nonzero value (*P* = 0.1). The positive median slope of the medium-resolution data did not significantly differ from zero (*P* = 0.4). In contrast, in the Aβ+ group, all median slopes of low-resolution (*P* < 0.05), medium-resolution (*P* < 0.05), and high-resolution (*P* < 0.05) data were significantly larger than zero; the median slopes of the medium- and high-resolution reconstructions were greater than the median slope of the low-resolution reconstructions. However, only the median slope of high-resolution reconstructions trended toward a significantly larger value (*P* = 0.08) than the median slope of low-resolution reconstructions. Thus, high- and medium-resolution reconstructions produced more positive slopes than the low-resolution reconstruction.

We turn now to an analysis of the relative median accumulation slopes between low-, medium- and high-resolution reconstructions in both the Aβ− and the Aβ+ groups. In the Aβ− group, the median accumulation slope of high-resolution reconstruction data was significantly larger than the low-resolution case (*P* = 0.03), and the median accumulation slope of medium-resolution data trended toward a larger value (*P* = 0.07) than the low-resolution case (Fig. 3C). Notably, the difference in median accumulation slopes was not seen in the Aβ+ group, except for a trend toward a significant difference between high- and low-resolution reconstructions (*P* = 0.06) (Fig. 3D).

The number of Aβ accumulators was calculated as a third comparison measure of reconstruction configurations. Medium- and high-resolution reconstructions identified 10 additional Aβ accumulators in the Aβ− group compared with low-resolution reconstruction data when the threshold was 0 CL/y; higher-resolution reconstructions showed a 22% increase in identifying Aβ accumulators. However, equal number of Aβ accumulators were identified in the Aβ− group with the threshold of 2 CL/y. There was no notable difference in the number of accumulators in the Aβ+ group for a threshold of either 0 or 2 CL/y (Supplemental Table 2). In summary, medium- and high-resolution data identified more Aβ accumulators in the Aβ− group.

## DISCUSSION

This study examined the impact of PET image spatial resolution, determined by the reconstruction protocol, on Aβ quantitation in both cross-sectional and longitudinal datasets. The choice of reconstruction algorithm and parameter settings is known to govern spatial resolution, which impacts Aβ quantitation via the PVE (14*,*^21^). Accuracy of Aβ measures has implications both clinically and preclinically, affecting diagnosis and staging of AD, monitoring of disease progression, tailoring of treatments for personalized medicine, and AD clinical trials (1*,*^2^).

The spatial resolution of reconstruction protocols, quantified by phantom-derived FWHMs, impacted the CLs of cross-sectional data for both the Aβ− and the Aβ+ groups. This may be due to the PVE in the white matter (WM) and gray matter (GM) regions of the brain; whereas current Aβ PET radiotracers are designed to bind to GM structures, some nonspecific binding occurs, particularly in WM regions (2). Aβ− subjects tend to show higher tracer retention in WM than GM, whereas Aβ+ subjects tend to shown higher tracer retention in GM than WM (22). In Aβ− subjects, PVE may cause high-activity WM regions to contaminate GM activity, resulting in artificially increased CL estimates. The reverse effect from PVE is likely to occur in the Aβ+ group, with a dilution of GM activity from adjacent lower-activity tissue. The impact of PVE will change with the spatial resolution of the reconstruction protocol, and so too will CL quantitation.

Our cross-sectional analysis demonstrated that although the application of smoothing changed the FWHM of the reconstruction, it did not have a significant effect on the CLs in the Aβ− group. In contrast, there was a marked CL reduction in the Aβ+ group after postreconstruction gaussian smoothing. We conclude that postreconstruction smoothing may hinder CL quantitation by yielding differential effects on Aβ− and Aβ+ groups. Past studies have also suggested that smoothing may reduce or remove small differences due to pathology (23). Therefore, it is worth reconsidering scanner harmonization methods that use postreconstruction smoothing to match the spatial resolution as a mitigation of multicenter PET quantitation differences (24*,*^25^). A possible alternative for harmonizing scanners is to match the nonsmoothed FWHMs between scanners by adjusting reconstruction parameters, such as the number of iterations and subsets. Further studies are needed to examine the potential of nonsmoothed FWHM matching for multiscanner harmonization.

We found that cross-sectional CLs are impacted by algorithm convergence, determined by the product of subsets and iterations. This is expected given that algorithm convergence is a primary factor determining the spatial resolution, with more converged reconstructions resulting in higher spatial resolution, irrespective of the reconstruction algorithm being used (6*,*^8^).

The choice of reconstruction algorithm, specifically the inclusion or exclusion of PSF and TOF, impacted CL quantitation. Inclusion of a PSF is known to produce images of higher spatial resolution (4*,*^11^*,*^12^). This resulted in a reduced PVE, and consequently the PSFTOF reconstructions gave smaller and greater CLs in the Aβ− and Aβ+ groups than did OP and OPTOF. Moreover, the OPTOF and OP algorithms demonstrated similar spatial resolutions with increased iteration updates, suggesting the potential for comparable CL quantification in the Aβ− group at higher iteration updates. However, CLs were lower for OPTOF than for OP in the Aβ+ group. The incorporation of TOF technology enhances the signal-to-noise ratio in PET images (10). One potential rationale is that the increased signal-to-noise ratio might amplify the dilution effect on GM activity from neighboring tissues with lower activity in the Aβ+ group, thus lowering the CLs in OPTOF reconstructions; further studies are needed to elucidate the underlying reasons for this observed behavior.

Although we hypothesized that the spatial resolution of the reconstruction configuration impacts CL quantitation, the CLs of the Aβ− groups were not notably impacted by postreconstruction smoothing. One possible explanation is that postreconstruction smoothing may be affected by the number of coincidence events detected at the PET detectors; these counted events were notably lower in the Aβ− group than in the Aβ+ group. However, the reason for this phenomenon is unknown and needs to be examined in further studies.

High-resolution reconstructions resulted in a larger separation between the Aβ− and Aβ+ groups; cross-sectional analysis showed that differences between these groups decreased with increasing FWHM. This was a consequence of the opposing CL trend in the 2 groups, with CLs increasing in the Aβ− group and decreasing in the Aβ+ group with FWHM. This is in contrast to a study that reported similar Cohen *d* values irrespective of the reconstruction method (17); however, it is notable that a smaller cohort and a more limited range of reconstruction configurations were used, along with a different Aβ PET radiotracer. Moreover, our cross-sectional results showed that reconstruction configurations with better resolution gave the minimum and maximum CLs in the Aβ− and Aβ+ groups, respectively, while increasing the dynamic range of CLs in both groups; this may be due to the inherently lower PVE associated with high-resolution reconstructions, enabling a more accurate depiction of the underlying tracer distribution. Consequently, we believe that the higher-resolution reconstructions may capture the true interindividual CL differences. These results suggest that we should reexamine the use of modern reconstruction configurations with better spatial resolution as a standard reconstruction protocol in Aβ PET neuroimaging.

To the best of our knowledge, this was the first study examining the impact of spatial resolution on longitudinal Aβ PET data. As shown by the ARD metric, we found that Aβ accumulation across the longitudinal time course was more consistent for higher-resolution reconstructions than for lower-resolution reconstructions, particularly in the Aβ− group. This is a clinically important result, as longitudinal Aβ PET data should match the assumed linearity property of the Aβ accumulation process over a short time interval. However, as shown by the ARD metric, more consistent Aβ accumulation was not seen in the Aβ+ group for all the reconstructions; this observation may be attributed to the dependence of the Aβ accumulation rate on the level of Aβ such that the linearity assumption may be violated. Moreover, it is crucial to the clinical management of early-stage AD therapies that Aβ accumulators be identified at low Aβ levels, as AD prevention therapies require testing at early AD stages. We have demonstrated a 22% increase in the number of Aβ accumulators identified in the Aβ− group when using higher-resolution reconstruction configurations than when using the clinical low-resolution reconstruction configuration.

A limitation of the current study is that data from a single scanner and reconstruction toolbox were used. Although the impact of reconstruction parameters will show the same trend on other scanners, such as the HRRT (Siemens), Biograph Vision (Siemens), and uExplorer (United Imaging), the significance of the impact may differ because of factors such as scanner sensitivity, TOF resolution, and hardware-induced spatial resolution. Additional limitation are that reconstruction using different software and artifact corrections may also produce different results, only 2 Aβ PET radiotracers were used, and the duration of the longitudinal data spanned approximately 3 y. The results may depend on the radiotracer, as the nonspecific binding may change across tracers. Irrespective of these limitations, we found that high-resolution, converged reconstructions were better for Aβ PET quantitation than low-resolution reconstructions, in both cross-sectional and longitudinal studies. Future studies can be conducted to examine the feasibility of harmonization between scanners by matching the barrel phantom–derived spatial resolutions of reconstruction methods.

## CONCLUSION

High-resolution reconstructions, with inherently less PVE, can improve both cross-sectional and longitudinal Aβ PET data quantitation as demonstrated by the increased separation between the Aβ− and Aβ+ groups in the cross-sectional analysis and more consistent Aβ accumulation in longitudinal data than is possible with low-resolution scans, in conjunction with the identification of more Aβ accumulators in the Aβ− group. These Aβ PET quantitation improvements gained from high-resolution reconstructions are an important aspect of understanding AD progression and the management of early-stage AD therapies using PET imaging. Our results demonstrate that postreconstruction smoothing may hinder CL quantitation, suggesting that the use of postreconstruction smoothing as a scanner harmonization method may not fully achieve its intended outcome.

## DISCLOSURE

The research was supported by the Australian Federal Government through NHMRC and NIH grants. No other potential conflict of interest relevant to this article was reported.

## Notes

KEY POINTS **QUESTION:** Does PET spatial resolution, as determined by the choice of reconstruction configuration, affect the quantitation of cross-sectional and longitudinal Aβ PET? **PERTINENT FINDINGS:** In the cross-sectional analysis, high-resolution reconstructions gave the lowest and highest Aβ CLs in Aβ− and Aβ+ groups, respectively, improving the separation between the groups. High-resolution reconstructions improved the longitudinal consistency of measured Aβ accumulation and was more sensitive, identifying more Aβ accumulators. **IMPLICATIONS FOR PATIENT CARE:** High-resolution scans can improve both cross-sectional and longitudinal Aβ PET data quantitation, which will assist in understanding AD progression and management of AD therapies in the early stages.
